# Functional analysis of the Bunyamwera orthobunyavirus Gc glycoprotein

**DOI:** 10.1099/vir.0.013540-0

**Published:** 2009-10

**Authors:** Xiaohong Shi, Josthna Goli, Gordon Clark, Kristina Brauburger, Richard M. Elliott

**Affiliations:** Centre for Biomolecular Sciences, School of Biology, University of St Andrews, North Haugh, St Andrews KY16 9ST, Scotland, UK

## Abstract

The virion glycoproteins Gn and Gc of Bunyamwera orthobunyavirus (family *Bunyaviridae*) are encoded by the M RNA genome segment and have roles in both viral attachment and membrane fusion. To investigate further the structure and function of the Gc protein in viral replication, we generated 12 mutants that contain truncations from the N terminus. The effects of these deletions were analysed with regard to Golgi targeting, low pH-dependent membrane fusion, infectious virus-like particle (VLP) formation and virus infectivity. Our results show that the N-terminal half (453 residues) of the Gc ectodomain (909 residues in total) is dispensable for Golgi trafficking and cell fusion. However, deletions in this region resulted in a significant reduction in VLP formation. Four mutant viruses that contained N-terminal deletions in their Gc proteins were rescued, and found to be attenuated to different degrees in BHK-21 cells. Taken together, our data indicate that the N-terminal half of the Gc ectodomain is dispensable for replication in cell culture, whereas the C-terminal half is required to mediate cell fusion. A model for the domain structure of the Gc ectodomain is proposed.

## INTRODUCTION

Viruses in the family *Bunyaviridae* (known as bunyaviruses) are mainly arthropod-transmitted and are classified into five genera: *Orthobunyavirus*, *Hantavirus*, *Nairovirus*, *Phlebovirus* and *Tospovirus*. Several bunyaviruses are important human pathogens causing emerging zoonotic infections, such as La Crosse (LACV), Hantaan (HTNV), Sin Nombre (SNV), Rift Valley fever (RVFV) and Crimean–Congo hemorrhagic fever (CCHFV) viruses. The characteristic features of bunyaviruses include similarity in virion morphology, possession of a tripartite, single-stranded RNA genome of negative- or ambisense polarity, cytoplasmic site of virus replication, and assembly and budding occurring at membranes of the Golgi complex (Elliott *et al.*, 2000; Nichol *et al.*, 2005). *Bunyamwera virus* (BUNV) is the prototype of both the family *Bunyaviridae* and the genus *Orthobunyavirus*.

Orthobunyaviruses encode two glycoproteins called Gn and Gc that form spikes on the virus particle and are involved in viral attachment and cell fusion (Schmaljohn & Hooper, 2001). They are encoded by the medium (M) RNA genome segment as a precursor protein (Gn–NSm–Gc), which is cleaved co-translationally to yield the two mature glycoproteins and a non-structural protein termed NSm (Elliott, 1996). Both Gn and Gc are type I integral transmembrane (TM) proteins and are modified by *N*-linked glycosylation (Nichol *et al.*, 2005; Schmaljohn & Hooper, 2001; Shi *et al.*, 2005). The BUNV Gn protein consists of 302 residues with a rather long predicted cytoplasmic tail (CT) of 78 residues, whilst its larger counterpart Gc encompasses 957 residues with a shorter CT of 25 residues (Elliott, 1990; Lees *et al.*, 1986). The two proteins form a heterodimer and Gn plays a chaperone-like role to promote Gc folding and Golgi targeting (Shi *et al.*, 2004, 2005).

It is generally accepted that both glycoproteins are required for virus entry, but little is known about the involvement of the individual glycoproteins in the early events of virus infection. Studies on LACV implicated Gc as the viral attachment protein for both insect and mammalian cells (Hacker *et al.*, 1995; Kingsford *et al.*, 1983; Sundin *et al.*, 1987), although other data suggest Gc as the attachment protein for mammalian cells and Gn for mosquito cells (Ludwig *et al.*, 1991). However, as two non-temperature-sensitive (ts) revertants (R1 and R2) of a ts mutant of Maguari virus (MAGV, also an orthobunyavirus) contain considerable deletions in the N terminus of Gc (R1 has 239 residues deleted and R2 has 431 aa missing; Elliott *et al.*, 1984; Murphy & Pringle, 1987; Pollitt *et al.*, 2006), the N-terminal region of the larger glycoprotein is not required for infection of, or replication in, cultured mammalian cells.

The glycoproteins of most enveloped viruses are also fusogenic in order to deliver the viral genome into the cytoplasm to initiate virus replication (Harrison, 2005, 2008; Weissenhorn *et al.*, 2007). The glycoproteins of several bunyaviruses, such as LACV and BUNV (orthobunyaviruses) (Jacoby *et al.*, 1993; Plassmeyer *et al.*, 2005; Shi *et al.*, 2007), RVFV (phlebovirus) (Filone *et al.*, 2006), HTNV (hantavirus) (Ogino *et al.*, 2004) and tomato spotted wilt tospovirus (TSWV) (Whitfield *et al.*, 2005), are reported to mediate acidic pH-dependent cell fusion. Bioinformatic proteomic analyses suggested that the Gc proteins of bunyaviruses share several sequence and structure-motif similarities with envelope protein I of Sindbis virus, a class II fusion protein (Garry & Garry, 2004). Recent data on LACV and hantavirus glycoproteins support the notion that Gc functions as the fusion protein (Plassmeyer *et al.*, 2005, 2007; Tischler *et al.*, 2005). However, as mutations in the CT of BUNV Gn affect membrane fusion severely (Shi *et al.*, 2007), Gn must also play an important role in the fusion process.

We constructed 12 glycoprotein precursor mutants that contain progressive deletions in the N terminus-encoding region of the BUNV Gc protein and investigated the role of the Gc ectodomain in virus replication with regard to Golgi targeting, fusogenicity, virus-like particle (VLP) formation and virus infectivity. Our data reveal that the N-terminal half of the Gc ectodomain is dispensable for intracellular Golgi targeting and low pH-induced cell fusion.

## METHODS

### Cells and viruses.

BHK-21 and BSR-T7/5 (Buchholz *et al.*, 1999) cells were maintained in Glasgow minimal essential medium (Invitrogen) supplemented with 10 % tryptose phosphate broth, 10 % fetal calf serum and, for BSR-T7/5 cells only, 1 mg geneticin ml^−1^ (G418 sulfate; Calbiochem) as described previously (Lowen *et al.*, 2004; Shi & Elliott, 2004). Working stocks of wild-type (wt) BUNV were grown in BHK-21 cells and titres were determined by plaque assay as described previously (Watret *et al.*, 1985).

### Antibodies.

A rabbit antiserum against purified BUNV virions (anti-BUN) has been described previously (Lappin *et al.*, 1994; Watret *et al.*, 1985). A mouse monoclonal antibody (mAb) against GM130, a *cis*-Golgi matrix protein (Nakamura *et al.*, 1995), was purchased from BD Biosciences. Goat anti-rabbit antibody conjugated with fluorescein isothiocyanate was purchased from Sigma and goat anti-mouse antibody conjugated with Cy5 was purchased from Chemicon International, Inc.

### Plasmids.

**Fig. 1. f1:**
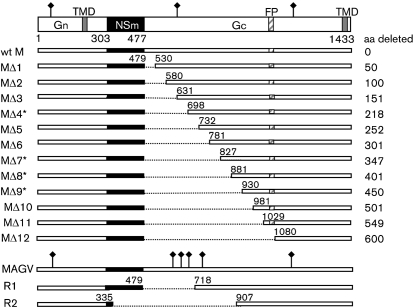
BUNV glycoprotein Gc mutants. The layout of the BUNV M segment-encoded gene product is shown at the top, with positions of amino acid residues marking protein boundaries (Gn, NSm and Gc) indicated. Below are shown schematics of the proteins encoded by wt and mutant (MΔ1, etc.) cDNA clones. Deletions start at residue 480 to keep intact the cleavage site between NSm and Gc. The N-terminal position of each mutant Gc protein and the number of residues (aa) deleted are shown. The M segment gene products of MAGV and its two non-ts revertant viruses R1 and R2 (Pollitt *et al.*, 2006) are shown beneath. Predicted transmembrane domains (TMD) and the fusion peptide (FP) in BUNV Gc (residues 1058–1079) are shown as grey and hatched boxes, respectively. ⧫ indicate glycosylation sites. Viruses recovered from mutant M segment cDNAs by reverse genetics are marked with an asterisk (*).

Plasmids that express BUNV proteins (pTM1-BUNL for expressing L protein, pTM1-BUNM for expressing Gn, NSm and Gc, and pTM1-BUNS for expressing N) or generate full-length antigenome RNA transcripts [pT7riboBUNL(+), pT7riboBUNM(+) and pT7riboBUNS(+)] have been described previously (Bridgen & Elliott, 1996; Lowen *et al.*, 2004), as has the BUNV-derived minigenome, pT7riboBUNMRen(−) (which contains the *Renilla* luciferase gene) (Weber *et al.*, 2001). pTM1-FF-Luc contains the firefly luciferase gene in the vector pTM1 (Weber *et al.*, 2002). Twelve BUNV M segment cDNA mutants that contain progressive deletions in the N terminus-encoding region of the Gc protein were derived from both pT7riboBUNM(+) and pTM1-BUNM by using a PCR mutagenesis approach (Shi & Elliott, 2002) (Fig. 1). All constructs were confirmed by DNA sequence analysis. The primers used and details of PCR are available upon request.

### Metabolic radiolabelling and immunoprecipitation.

Procedures for metabolic radiolabelling and immunoprecipitation of BUNV proteins were described previously (Shi *et al.*, 2004). Briefly, at 24 h post-transfection or at various time points after virus infection (as detailed in the figure legends), cells were labelled with [^35^S]methionine (80 μCi/2.96 MBq) for 1 h and then lysed on ice with either 150 μl (for direct SDS-PAGE analysis) or 300 μl (for immunoprecipitation) non-denaturing RIPA buffer [50 mM Tris/HCl (pH 7.4), 1 % Triton X-100, 300 mM NaCl, 5 mM EDTA] containing a cocktail of protease inhibitors (Roche). BUNV glycoproteins were immunoprecipitated with anti-BUN serum that had been conjugated to protein A–Sepharose (Sigma). The beads were washed with RIPA buffer containing 0.1 % Triton X-100 and once with ice-cold PBS, and the bound proteins were analysed by SDS-PAGE under reducing conditions.

### Indirect immunofluorescence staining.

Immunofluorescence assays were performed as described previously (Shi & Elliott, 2002). Briefly, transfected cells were fixed with 4 % paraformaldehyde and then permeabilized with 0.1 % Triton X-100 in PBS before staining with specific primary antibodies and secondary antibody conjugates. Localization of fluorescently labelled proteins was examined by using a Zeiss LSM confocal microscope.

### BUNV glycoprotein fusion assay.

The fusion assay was performed as described previously (Shi *et al.*, 2007). Briefly, BSR-T7/5 cells grown on 12-well plates were transfected with 1 μg of either pTM1-BUNM or one of the mutant M cDNAs. At 24 h post-transfection, cells were treated with low-pH medium (pH 5.3) for 5 min and then the medium was replaced by normal growth medium. Cell fusion was observed after further incubation for 4 h at 37 °C and Giemsa staining. Fusion was quantified by counting the number of cells and nuclei present in a microscopic field. Fusion index (*f*) was calculated according to the equation *f*=[1−(*c*/*n*)], where *c* is the number of cells in a field and *n* the number of nuclei (White *et al.*, 1981). An average field at a magnification of ×200 contained 400–600 nuclei. The mean *f* from three fields was calculated.

### Virus assembly assay.

The assay to measure infectious VLP production was performed as described previously with modifications (Shi *et al.*, 2006). Briefly, BSR-T7/5 cells grown in 12-well plates were transfected with a plasmid mixture containing pTM1-BUNS (0.05 μg), pTM1-BUNL (0.1 μg), pTM1-BUNM or one of the M cDNA mutants cloned in pTM1 (0.1 μg) and 0.2 μg of the BUNV-derived minigenome, pT7riboBUNMRen(−), together with 0.05 μg pTM1-FF-luc as internal control. At 24 h post-transfection, the supernatant was used to infect fresh BSR-T7/5 cells that had previously been transfected with pTM1-BUNS (0.05 μg) and pTM1-BUNL (0.1 μg). *Renilla* luciferase activity was measured after a further 24 h incubation by using a Dual-Luciferase Assay kit (Promega) according to the manufacturer's instructions.

### Virus rescue by reverse genetics and virus growth curves.

Rescue experiments were performed as described previously (Lowen *et al.*, 2004). Briefly, BSR-T7/5 cells were transfected with a mixture of three plasmids: 1.0 μg each of pT7riboBUNL(+), pT7riboBUNS(+) and either pT7riboBUNM(+) or one of the mutant M cDNAs cloned in pT7ribo. At 5 h post-transfection, 4 ml growth medium was added and incubation was continued for 5–11 days at 33 °C. Transfectant viruses were isolated by plaque formation on BHK-21 cells as described previously (Watret *et al.*, 1985). For virus growth curves, BHK-21 cells in 35 mm diameter Petri dishes were infected at an m.o.i. of 0.01 p.f.u. per cell and supernatants were harvested at various times after infection. Virus titres were determined by plaque formation on BHK-21 cells.

## RESULTS

### Generation and expression of mutant BUNV glycoproteins containing deletions in the N terminus of Gc

The complete BUNV Gc protein contains 957 residues, comprising the ectodomain of 909 residues, a TM domain of 23 residues and a relatively short CT of 25 residues (Fig. 1). The TM domain was predicted by using the program tmhmm (http://www.cbs.dtu.dk/services/TMHMM/; Moller *et al.*, 2001). To study the structure and function of the Gc ectodomain in the virus life cycle, we generated 12 mutant BUNV M segment cDNAs that contained truncations ranging from 50 to 600 amino acid residues in the N terminus-encoding region of Gc by PCR-directed mutagenesis (Fig. 1). To ensure that correct cleavage between Gc and the upstream NSm protein in the precursor occurred at the conserved Ala residue (A477; Elliott, 1990), the first three N-terminal residues of Gc (A477, E478 and E479) were retained in all deletion constructs. These three residues are also maintained in the naturally occurring MAGV R1 mutant that has a large deletion at the N terminus of Gc (Pollitt *et al.*, 2006).

**Fig. 2. f2:**
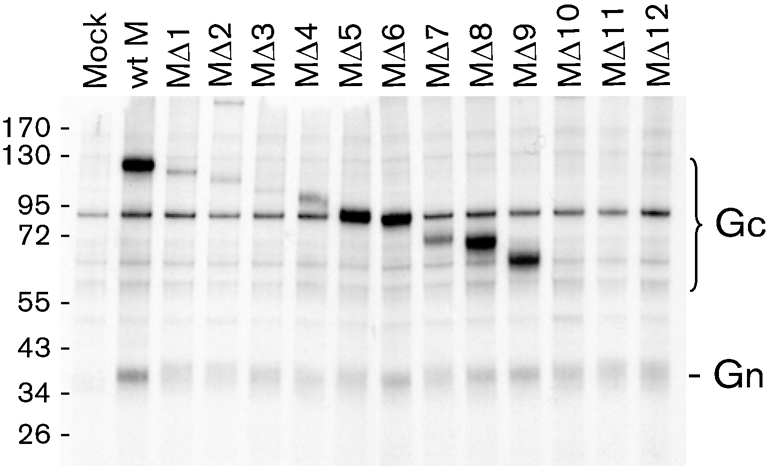
Proteins expressed from mutant BUNV M segment cDNAs. BSR-T7/5 cells were transfected with cDNAs as indicated or left untransfected (Mock) and labelled with [^35^S]methionine for 1 h at 24 h post-transfection, and cell lysates were reacted with anti-BUN serum. Immunoprecipitated proteins were analysed on 4–12 % polyacrylamide NuPAGE gels (Invitrogen) under reducing conditions. The positions of glycoproteins Gn and Gc and of protein molecular mass markers (in kDa) are indicated.

The mutant cDNA constructs were transfected into BSR-T7/5 cells and the expressed proteins were analysed by immunoprecipitation with anti-BUN antibodies and SDS-PAGE (Fig. 2). Gc and Gn proteins were clearly identified in cells transfected with the wt M segment cDNA (lane 2). Progressive deletion of the N terminus of Gc resulted in reduction in size of the Gc band in cells transfected with mutants MΔ1–MΔ9. No Gc band could be identified clearly in cells transfected with constructs MΔ10, MΔ11 or MΔ12 (containing the largest deletions). However, Gn was identified in all transfected cells, indicating that expression and processing of the precursor protein did occur for all constructs. This suggests that the failure to detect Gc for mutants MΔ10–MΔ12 was probably due to loss of epitopes recognizable by anti-BUN serum. It was noticeable that the truncated Gc proteins expressed by MΔ1, MΔ2 and MΔ3 were obviously weaker than the Gc bands of wt M or the other mutants, which may indicate a problem with efficient expression or correct folding of these proteins.

### Intracellular localization of truncated BUNV glycoproteins

Golgi trafficking and retention are characteristic of BUNV glycoproteins and depend on proper folding of, and heterodimer formation between, Gn and Gc. Maturation and Golgi targeting of Gc are dependent on co-expressed Gn that harbours the Golgi-targeting and -retention signal in its TM domain (Lappin *et al.*, 1994; Shi *et al.*, 2004, 2005). To examine the effect of N-terminal deletions of Gc on protein folding and intracellular trafficking, we compared the intracellular location of the mutant proteins by immunofluorescence, using a specific *cis*-Golgi matrix marker, GM130 (Nakamura *et al.*, 1995), in colocalization studies. Unfortunately, none of the mutant Gc proteins reacted with the available Gc-specific mAbs 742 and 810 (data not shown); these antibodies recognize an epitope close to the first glycosylation site in Gc (residue N624; Shi *et al.*, 2005). Therefore, cells transfected with mutant cDNAs were reacted with anti-BUN serum, which detects both Gn and Gc glycoproteins. This hampered interpretation somewhat, as Gn by itself is able to target the Golgi, albeit less efficiently than when co-expressed with functional Gc (Lappin *et al.*, 1994; Shi *et al.*, 2004, 2005).

**Fig. 3. f3:**
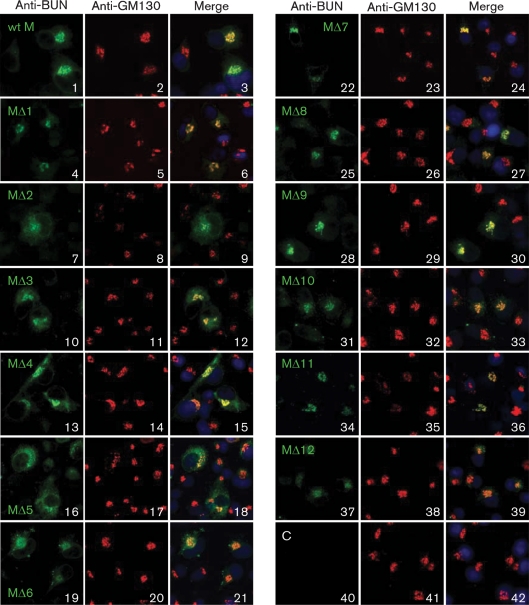
Intracellular localization of BUNV glycoproteins. BSR-T7/5 cells were transfected with wt or mutant BUNV M segment cDNAs as indicated or left untransfected (C), and stained with a mixture of rabbit anti-BUN and mouse anti-GM130 antibodies. BUNV glycoproteins Gn and Gc stain green and GM130 stains red. In merged images, the colocalization of BUNV glycoprotein and GM130 shows as yellow. Nuclei were stained blue with 4′,6-diamidino-2-phenylindole (DAPI).

Of the 12 mutant BUNV M segment cDNA constructs, the staining patterns of four mutants (MΔ1, MΔ7, MΔ8 and MΔ9) showed typical Golgi localization similar to that of the wt BUNV M cDNA control, indicating that the truncated Gc proteins expressed from these constructs were able to target to the Golgi complex efficiently (Fig. 3, compare panel 3 with panels 6, 24, 27 and 30). The glycoproteins expressed by MΔ4 were predominantly localized in the Golgi, although cytoplasmic staining was also visible (panels 13–15). The glycoproteins expressed by MΔ2, MΔ3, MΔ5 and MΔ6 showed mainly a cytoplasmic staining pattern, suggesting that the mutations in these constructs affected the protein folding and/or interactions required for Golgi targeting. The glycoproteins expressed by MΔ10–MΔ12 showed rather weaker staining, partially explainable by the reduced reactivity of the truncated Gc proteins with anti-BUN serum. The Golgi colocalization observable in cells transfected with constructs MΔ3, MΔ5, MΔ6 and MΔ10–MΔ12 is, we suggest, probably detection of Gn in this organelle. These results showed that the impact of the deletions on the ability of Gc to traffic to the Golgi was not linearly proportional to the number of residues removed, but probably reflected incorrect folding of the expressed protein. For instance, Gc proteins expressed by MΔ2 and MΔ3, with only 100 and 151 residues deleted respectively, failed to transport to the Golgi, whereas Gc proteins expressed by MΔ7, MΔ8 and MΔ9, with deletions of between 347 and 450 residues, were still competent for Golgi targeting and retention. Furthermore, our observations indicated that nearly half (49.5 %) of the N-terminal Gc ectodomain (as deleted in MΔ9) is not required for Golgi trafficking.

### Requirement of the Gc N terminus in low pH-induced cell fusion

**Fig. 4. f4:**
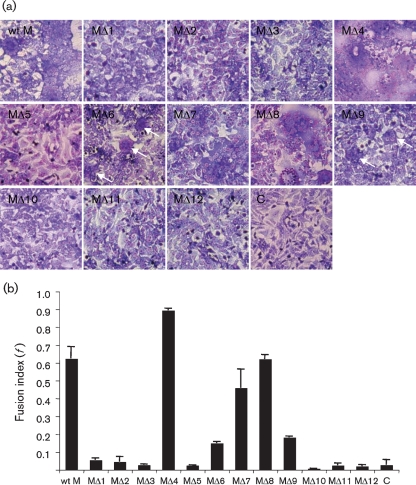
Low pH-induced syncytium formation. BSR-T7/5 cells were transfected with wt or mutant BUNV M segment cDNA constructs or left untransfected as a control (C). At 24 h post-transfection, cells were treated with low-pH medium (pH 5.3) for 5 min and syncytium formation was examined following incubation at 37 °C for a further 5 h. Cells were then stained with Giemsa solution. (a) Syncytium formation in cells transfected with wt (wt M) and mutant BUN M segment cDNAs (MΔ1–MΔ12) as indicated. The small syncytia formed by cells transfected with MΔ6 and MΔ9 cDNAs are marked by white arrows. (b) Fusion indices (*f*), calculated as described in Methods from cells treated with low-pH medium.

Low-pH treatment of either BUNV-infected or M cDNA-transfected cells leads to cell fusion (Shi *et al.*, 2007). To investigate the involvement of the N terminus of Gc in cell fusion, BSR-T7/5 cells transfected with the mutant cDNAs were treated with low-pH medium (pH 5.3) and syncytium formation was quantified to estimate the fusion index. As shown in Fig. 4(a), cell fusion was observed in cells transfected with five mutant cDNAs (MΔ4 and MΔ6–MΔ9). Large syncytia were seen in cells transfected with MΔ4, MΔ7 and MΔ8, whereas smaller syncytia were observed in cells transfected with MΔ6 and MΔ9 (marked by arrows). No syncytium formation was seen in cells transfected with the other seven constructs. Similar to the effect on Golgi trafficking, the impact on cell-fusion activity did not correlate with the number of amino acids deleted. Deletions of 50, 100 and 151 residues (as in constructs MΔ1–MΔ3, respectively) abrogated fusion activity, whereas deletion of a further 67 residues (construct MΔ4) restored the fusogenic activity of the expressed glycoproteins. In fact, MΔ4, which has a deletion of 218 residues at the N terminus of Gc, showed even higher fusogenic activity (*f*=0.894) than the wt control (*f=*0.623; Fig. 4b[Fig f4]). Removal of an additional 34 residues (construct MΔ5) again resulted in total loss of fusogenic activity, but the successive deletion of residues 732–880 (constructs MΔ5–MΔ8) resulted in proteins that displayed fusion activity to a level comparable to that of the wt control (Fig. 4b[Fig f4]). The cell fusion of construct MΔ9 was reduced significantly (*f*=0.181) and no syncytium formation was seen when deletions beyond residue 930 in the central ectodomain, i.e. close to or including the predicted fusion peptide, were made (constructs MΔ10–MΔ12) (Fig. 4a, b[Fig f4]). Our data suggest that the N-terminal 401 residues are not involved in the fusion process, whereas the central region of the Gc ectodomain from residues 930 to 1080 (constructs MΔ9–MΔ12) is needed for fusion to occur.

### Role of the Gc N terminus in virus particle assembly and viability

**Fig. 5. f5:**
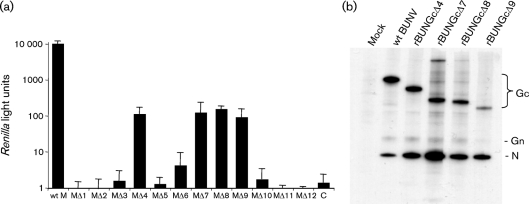
Effects of deletions in the N terminus of the Gc ectodomain on virus assembly. (a) VLP formation. BSR-T7/5 cells were transfected with minigenome component plasmids together with either wt or mutant M segment cDNA constructs. Supernatants from these cells were used to infect new BSR-T7/5 monolayers previously transfected with BUNV L and N protein-expressing plasmids. *Renilla* luciferase activities were measured after a further 24 h incubation and are shown as arbitrary light units. (b) Protein profiles of the rescued mutant viruses. BHK-21 cells were infected with either wt BUNV or mutant viruses and cells were labelled with [^35^S]methionine for 1 h at 24 h post-infection. Cell lysates were analysed on 4–12 % polyacrylamide NuPAGE gels under reducing conditions. The positions of viral proteins are marked.

The effect of N-terminal deletions on virus assembly was assessed by the formation of infectious VLPs that package a genome analogue encoding the *Renilla* luciferase gene (Shi *et al.*, 2006). As shown in Fig. 5(a), infectious VLPs were produced in cells transfected with constructs MΔ4, MΔ7, MΔ8 and MΔ9. However, VLP formation was reduced significantly to approximately 1 % of that of the wt control. VLP formation was just detectable in cells transfected with MΔ6 (a reflection of the sensitivity of the assay), whereas no VLPs were made in cells transfected with the other constructs. Consistent with the results above for the VLP-formation assay, we were able to recover by reverse genetics four viable recombinant viruses from Gc deletion constructs MΔ4, MΔ7, MΔ8 and MΔ9 (the mutant viruses were designated rBUNGcΔ4, rBUNGcΔ7, rBUNGcΔ8 and rBUNGcΔ9). Analysis of the protein profiles of cells infected with the mutant viruses by SDS-PAGE showed that the viruses expressed smaller Gc proteins corresponding to the deletions introduced into the cDNA clones (Fig. 5b[Fig f5]).

### Effect of deletions in the N terminus of Gc on virus replication

**Fig. 6. f6:**
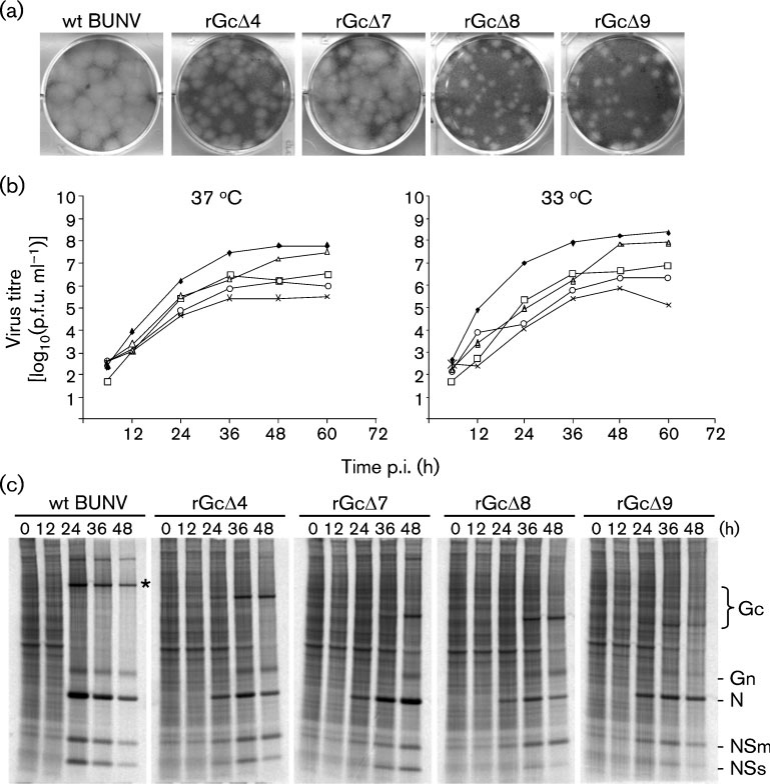
Plaque phenotypes, growth kinetics and protein synthesis profiles of wt and mutant viruses. (a) Comparison of plaque size on BHK-21 cells. Monolayers were fixed 6 days post-infection with 4 % formaldehyde and stained with Giemsa solution. (b) Virus growth curves in BHK-21 cells (at 37 and 33 °C). Cells were infected with either wt (⧫) or recombinant (□, rBUNGcΔ4; ▵, rBUNGcΔ7; ○, rBUNGcΔ8; ×, rBUNGcΔ9) viruses at an m.o.i. of 0.01 p.f.u. per cell. Virus was harvested at intervals as indicated and titrated by plaque formation in BHK-21 cells. Results are shown as the mean of two independent titrations. (c) Time-course of protein synthesis in infected BHK-21 cells. Cells were infected at an m.o.i. of 0.01 p.f.u. per cell and were labelled with 80 μCi [^35^S]methionine for 1 h at the time points indicated. Cell lysates were analysed on 4–12 % polyacrylamide NuPAGE gels. The positions of viral proteins are indicated; * indicates wt Gc.

The availability of four mutant viruses enabled us to investigate further the role of the N terminus in virus replication by comparing plaque size, growth kinetics and ability to shut off host protein synthesis. In BHK-21 cells, the four mutant viruses made plaques smaller than those of wt BUNV; in particular, rBUNGcΔ8 and rBUNGcΔ9 produced the smallest plaques (Fig. 6a[Fig f6]). Virus growth kinetics were compared in BHK-21 cells at two temperatures following low-multiplicity infection (i.e. multi-step growth cycle; Fig. 6b[Fig f6]). Recombinant viruses rBUNGcΔ4, rBUNGcΔ8 and rBUNGcΔ9 were attenuated, reaching titres 10- to 100-fold lower than wt BUNV at 37 °C. The differences were greater at 33 °C, with rBUNGcΔ9 showing marked attenuation, growing 1000-fold less well than wt BUNV. In contrast, rBUNGcΔ7, although growing more slowly, reached almost similar titres to wt BUNV at both temperatures by 48 or 60 h. The ability of the mutant viruses to induce shut-off of host-cell protein synthesis in BHK-21 cells reflected their slower growth rate compared with wt BUNV when the cells were infected at an m.o.i. of 0.01 p.f.u. per cell: wt BUNV achieved almost complete shut-off by 36 h post-infection, whereas the mutant viruses showed only minimal host shut-off at 48 h post-infection. Taken together, our data indicated that although all of the mutant viruses were able to infect mammalian cells, they were attenuated, suggesting that the Gc ectodomain is indeed required for efficient replication in BHK-21 cells.

## DISCUSSION

Infection of cells by enveloped viruses is initiated by the release of the virus genome into the replication compartment through the fusion of viral and cellular membranes, a process that is mediated by the viral fusion protein(s) (Harrison, 2008; Kielian & Rey, 2006; Weissenhorn *et al.*, 2007). Our results revealed that the N-terminal region of the Gc ectodomain (up to residue 880) is not required for membrane fusion, in agreement with experimental data for LACV Gc (Plassmeyer *et al.*, 2005, 2007) and bioinformatic predictions (Garry & Garry, 2004) suggesting that the orthobunyavirus fusion peptide is located in the membrane-proximal region. The fusion peptide of BUNV is predicted to encompass residues 1058–1079 of Gc (Garry & Garry, 2004) and is highly conserved among orthobunyaviruses (Cortez *et al.*, 2002). Indeed, mutagenesis of the analogous region in an LACV Gc cDNA clone followed by transient expression supported the notion that this domain functions as the fusion peptide (Plassmeyer *et al.*, 2007). Our data indicate that the residues flanking the fusion peptide are structurally critical for the conformational change during the fusion process: deletion in the region close to the fusion peptide (in constructs MΔ10–MΔ11) led to abolition of cell fusion.

Membrane fusion mediated by the flavivirus E protein is initiated by the protonation of specific histidine residues near the fusion protein (Fritz *et al.*, 2008). There are five histidine residues conserved in orthobunyavirus Gc ectodomains (at positions 797, 949, 1029, 1145 and 1295 in BUNV Gc), three of which are in the vicinity of the predicted fusion peptide. The first consensus histidine residue (H797) seems not to be involved in the fusion process, as deletion of this residue did not affect cell fusion (constructs MΔ7 and MΔ8). It would be of interest to mutate the other histidine residues specifically to ascertain their importance. It is usually the case that the N-terminal region of a fusion protein (which often contains the receptor-binding domain) must be displaced to allow extension of the fusion peptide or fusion loop to target the cellular membrane following the conformational change (Harrison, 2008). With respect to BUNV Gc, removal of the N-terminal 151 residues (construct MΔ4) actually enhanced cell fusion, perhaps suggesting that deletion of these residues facilitates the conformational change required for cell fusion. Thus, the function of this region could be to mask or protect the fusion peptide, although further experimentation is required to investigate this possibility.

Most enveloped viruses enter cells by receptor-mediated endocytosis (Marsh & Helenius, 2006; Smith & Helenius, 2004). For orthobunyaviruses, the mechanism of how Gn and Gc function in virus entry has still to be clarified (Schmaljohn & Hooper, 2001). Ludwig *et al.* (1989, 1991) suggested LACV Gc to be the attachment protein for mammalian cells and Gn that for mosquito cells, whereas others have described Gc as the attachment protein not only for both mammalian and mosquito cells, but also for mosquitoes (Hacker & Hardy, 1997; Hacker *et al.*, 1995; Pekosz *et al.*, 1995; Plassmeyer *et al.*, 2005; Sundin *et al.*, 1987). Our data presented above corroborate previous results on MAGV (Pollitt *et al.*, 2006) and indicate that the N-terminal domain of Gc of viruses in the Bunyamwera serogroup is not essential for infection of, and replication in, cultured cells. However, the significant reduction in VLP formation and attenuation of the mutant viruses suggests that the N-terminal domain does play some role in the infection process, and it would be informative to investigate whether the mutants are impaired in their ability to infect natural hosts. The fact that the mutant virus rBUNGcΔ9, which lacks nearly half of the Gc ectodomain, is still able to infect BHK cells suggests that receptor-binding activity (either all or part) could reside in the C-terminal half of Gc or that the Gn protein could also act as a viral attachment protein, possibly via alternative viral receptor(s). It is well-known that many viruses use more than one type of receptor for their entry (Marsh & Helenius, 2006).

Bunyavirus glycoproteins are targeted to, and retained in, the Golgi complex, where viruses mature and bud. For BUNV, correct protein folding and heterodimer formation between Gn and Gc are prerequisites for Golgi trafficking of both proteins, with Gn containing the Golgi-targeting and -retention signal (Lappin *et al.*, 1994; Shi *et al.*, 2004, 2005, 2007). The efficient Golgi targeting of the mutant glycoproteins expressed by constructs MΔ4, MΔ7, MΔ8 and MΔ9 indicated that the N-terminal half of the Gc ectodomain (residues 477–929) is not required for proper folding or heterodimeric interaction with Gn, which are presumably undertaken by residues in the C-terminal half of the Gc protein (residues 930–1433).

**Fig. 7. f7:**
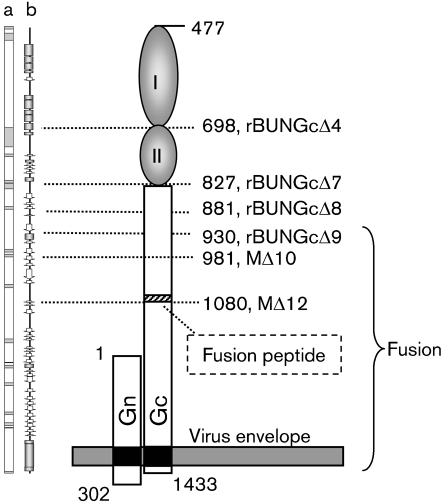
Model of the domain structure of the BUNV Gc protein. The lengths of BUNV glycoproteins Gn and Gc are drawn in proportion to their actual sizes. The prediction of disordered residues (a) and consensus secondary structure (b) of Gc, obtained by using the Phyre server (Kelley & Sternberg, 2009), are shown on the left. Disordered residues are indicated as shaded boxes in (a), and *α*-helices and *β*-strands are shown as shaded boxes and open arrows, respectively, in (b). The positions that indicate the residues of the N and C termini of Gn and Gc within the precursor protein, and residues defining the deletion mutations (MΔ4, MΔ7–MΔ10 and MΔ12), are shown. The predicted fusion peptide in Gc (residues 1058–1079) is indicated as a hatched box.

The analyses of the BUNV Gc ectodomain reported herein allow us to suggest a functional domain structure of the protein, in which the C-terminal half is essential for cell fusion, with the boundary lying between residues 930 and 982 (constructs MΔ9 and MΔ10). Assays for Golgi targeting, cell fusion and VLP formation are consistent with the N-terminal ectodomain comprising at least two structural domains (I and II; Fig. 7). Alignment of the model with the consensus secondary structure predicted by using the Phyre protein structure-ediction server (Kelley & Sternberg, 2009) revealed that domain I is mainly formed by a group of *α*-helices, whereas domain II is composed of coils, *β*-strands and two short *α*-helices (Fig. 7). Of note is that the regions linking domains I and II, and domain II with the downstream domain, are predicted to be disordered. Complete removal of either domain I (in the case of construct MΔ4) or domains I and II together (construct MΔ8) did not obviously affect the functions of Golgi targeting, fusogenicity or virus infectivity. However, deletions within domain I (constructs MΔ1–MΔ3) or within domain II (constructs MΔ5 and MΔ6) affected Gc function severely, suggesting that the remaining sequence that results from partial deletions within a domain would disrupt the overall structure, and thus functions, of the Gc protein. However, as the N-terminal region of Gc is dispensable for virus replication in tissue culture, it might be possible to insert foreign sequences, e.g. encoding an autofluorescent protein, in its place to generate recombinant BUNV expressing a tagged glycoprotein. Such a construct would be a valuable tool to investigate virus entry and budding processes, as has been achieved for other viruses (Brandenburg & Zhuang, 2007 and references therein).
